# Effect of the gut microbiome, skin microbiome, plasma metabolome, white blood cells subtype, immune cells, inflammatory proteins, and inflammatory cytokines on asthma: a two-sample Mendelian randomized study and mediation analysis

**DOI:** 10.3389/fimmu.2025.1436888

**Published:** 2025-03-21

**Authors:** Wenqian Guo, Er Hong, Han Ma, Ji Wang, Qi Wang

**Affiliations:** ^1^ School of Traditional Chinese Medicine, Southern Medical University, Guangzhou, China; ^2^ National Institute of Traditional Chinese Medicine Constitution and Preventive Medicine, Beijing University of Chinese Medicine, Beijing, China; ^3^ Department of Respiratory Medicine, Ningbo Hospital of Traditional Chinese Medicine, Zhejiang University of Chinese Medicine, Ningbo, China; ^4^ The Second Affiliated Hospital of Henan University of Chinese Medicine, Zhengzhou, China

**Keywords:** asthma, gut microbiota, skin microbiota, plasma metabolites, immune cells, inflammatory proteins, inflammatory cytokines, Mendelian randomization

## Abstract

**Background:**

Asthma is a chronic inflammatory disorder arising from incompletely understood heterogenic gene–environment interactions. This study aims to investigate causal relationships among gut microbiota, skin microbiota, plasma metabolomics, white blood cells subtype, immune cells, inflammatory proteins, inflammatory cytokines, and asthma.

**Methods:**

First, two-sample Mendelian randomization analysis was used to identify causal relationships. The summary statistics of 412 gut microbiota traits (N = 7 738), 150 skin microbiota traits (N = 579), 1 400 plasma metabolite traits (N = 8 299), white blood cells subtype counts (N = 746 667), 731 immune cell traits (N = 3 669), 91 circulating inflammatory proteins (N = 14 744), 41 inflammatory cytokine traits (N = 8 293), and asthma traits (N = 244 562) were obtained from publicly available genome-wide association studies. Inverse–variance weighted regression was used as the primary Mendelian randomization method. A series of sensitivity analyses was performed to test the robustness of causal estimates. Subsequently, mediation analysis was performed to identify the pathway from gut or skin microbiota to asthma mediated by plasma metabolites, immune cells, and inflammatory proteins.

**Results:**

Mendelian randomization revealed the causal effects of 31 gut bacterial features (abundances of 19 bacterial pathways and 12 microbiota), 10 skin bacterial features, 108 plasma metabolites (81 metabolites and 27 ratios), 81 immune cells, five circulating inflammatory proteins, and three inflammatory cytokines and asthma. Moreover, the mediation analysis results supported the mediating effects of one plasma metabolite, five immunophenotypes, and one inflammatory protein on the gut or skin microbiota in asthma pathogenesis.

**Conclusion:**

The findings of this study support a causal relationship among gut microbiota, skin microbiota, plasma metabolites, immune cells, inflammatory proteins, inflammatory cytokines, and asthma. Mediating pathways through which the above factors may affect asthma were proposed. The biomarkers and mediation pathways identified in this work provide new insights into the mechanism of asthma and contribute to its prevention and treatment.

## 1 Introduction

Asthma is a heterogeneous chronic respiratory disease defined by a history of respiratory symptoms (e.g., wheezing, shortness of breath, chest tightness, and coughing) that vary over time and in intensity together with variable expiratory airflow limitation. It affects nearly 10% of the global population, and its prevalence has continued to increase worldwide (1). Its prevalence, especially among children, has increased in numerous countries (2). This condition also presents challenges in terms of treatment efficacy and carries a substantial risk of exacerbation (3). Asthma imposes a significant disease burden, affecting all ages with increased mortality and reduced quality of life. Its pathogenesis, influenced by genetic and environmental factors, remains unclear, with genetic contributions estimated at 25%–80% (4). Given that asthma is a crucial public health concern associated with a high prevalence of morbidity and mortality, the study of its pathogenesis has certain importance.

The human gut microbiota is a dynamic ecosystem of microorganisms that interact with each other and the host. Dysbiosis not only impacts gut immunity but also influences lung health, contributing to the gut–lung axis concept (5). Gut microbiota influence lung immunity through immune cell differentiation and metabolite production affecting distal sites (6). The gut microbiota influences asthma susceptibility, with studies showing increased histamine-secreting microbes in asthma patients (7). In addition, research suggests that gut microbiota variation may help prevent or alleviate asthma by modulating inflammation, producing short-chain fatty acids, and regulating T cells (8). For example, the perturbed gut microbiota triggered by antibiotic use in individuals with asthma can be characterized by an exacerbated T helper (Th) 2 and Th1/Th17 immune response and diminished Treg population (9).

The mechanisms underlying the link between gut microbiota and asthma involve the complex metabolic and immune interactions of microbes, metabolites, and host immune responses (10). Moreover, gut microbiota is thought to play an important role in altering lung function, and several contributing pathways (Treg, iNKT, and Th17 cells) have been identified (6). Plasma metabolomics has identified key asthma-related metabolites (e.g., acetate, adenosine, alanine, and succinate) linked to hypoxia, oxidative stress, immunity, and lipid metabolism (11), with pediatric cases showing reduced citrate, ketone bodies, histidine, and glutamine (12), and aberrant purine metabolism in allergic asthma (13). Asthma arises from interactions between structural and immune cells triggered by environmental exposures (14), leading to airway obstruction mediated by chronic inflammation involving DCs, eosinophils, neutrophils, lymphocytes, ILCs, and mast cells. Altered microbiota disrupts immune homeostasis, impacting tolerance and inflammation (15). Airway inflammation drives hyperreactivity, mucus production, smooth muscle proliferation, and angiogenesis, worsening obstruction and lung function decline (16). Th2 CD4 activation promotes cytokine release (IL-4, IL-5, IL-13), leading to IgE synthesis, mast cell activation, and eosinophilic recruitment (17), with Th2–high inflammation commonly observed in allergic asthma (18).

Skin is colonized by diverse microbial communities and can be influenced over time in response to environmental factors. Skin microbiome plays an important role in tissue homeostasis and local immunity (19). One study found that Russian children had a higher abundance of Acinetobacter in their skin and nasal passages, which led to fewer cases of asthma and allergies, than Finnish individuals (20). A certain correlation exists between skin microbiota and environmental biodiversity in patients with allergies (21) Although the mechanistic role of skin dysbiosis in asthma pathogenesis remains uncertain, changes in the environment initiate dysbiosis in the skin along with the lung and gut, inducing functional and compositional changes in microbiota; such changes can affect the immunological mechanisms of allergic diseases, including asthma (22).

Observational studies make inferring true causality difficult given the presence of reverse causality and potential confounding factors. Mendelian randomization (MR) has a unique advantage in exploring the potential causal relationship between two traits on the basis of the Mendelian laws of inheritance and minimizes the effect of confounding factors on causal estimation (23). Mediation analysis can further evaluate the effects of an exposure on an outcome through a mediator (24). In contrast to airway microbiota, whose potential to modulate asthma is well recognized, gut and skin microbiota are anatomically distinct from the site of asthma occurrence but still play an important role in asthma. Meanwhile, the understanding of the effect of the skin microbiome on asthma is limited. In this study, we conducted a MR study based on recently published summary datasets of large genome-wide association studies (GWASs) to evaluate the causal relationship among gut microbiota, skin microbiota, plasma metabolites, circulating white blood cells (WBC), immune cells, circulating inflammatory proteins, inflammatory cytokines, and asthma and identify pathways from the gut or skin microbiota to asthma mediated by plasma metabolites, immune cells, and inflammatory proteins.

## 2 Methods

### 2.1 Study design

**Figure 1:**
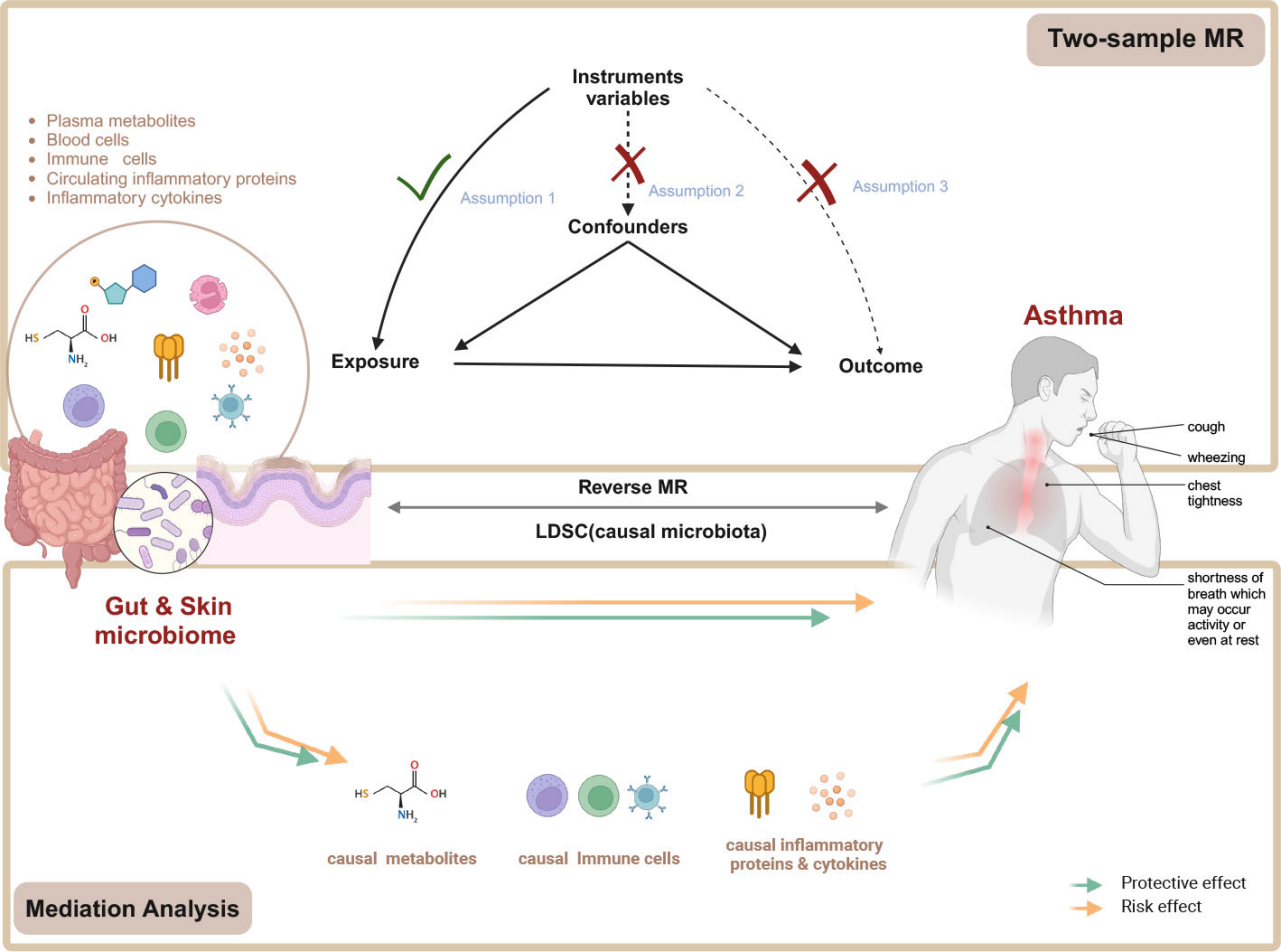
Flow chart of the study. Mendelian randomization study rationale: assumption 1, genetic instruments are associated with exposure; assumption 2, genetic instruments are not associated with confounders; assumption 3, genetic instruments are not associated with outcome, and genetic instruments act on outcome only through exposure. MR, Mendelian randomization. Created in BioRender. Wenqian, G. (2025) https://BioRender.com/w65a330.

The study flowchart is illustrated in **Figure 1**. First, published GWAS summary data that included traits, such as gut microbiota, skin microbiota, plasma metabolites, circulating WBC, circulating inflammatory proteins, inflammatory cytokines, and asthma, were obtained (**Supplementary Table S1**). Second, two-sample MR analyses were used to evaluate the causal relationship among gut microbiota, skin microbiota, plasma metabolites, circulating WBC, immune cell traits, circulating inflammatory proteins, inflammatory cytokines, and asthma. In addition, gut microbiota, skin microbiota, and asthma were further analyzed through bidirectional two-sample MR. Furthermore, linkage disequilibrium score regression (LDSC) was performed to identify causal microbiota and gut bacterial pathways. Finally, two-step analyses were used to identify the mediation effect of plasma metabolites, circulating inflammatory proteins, and inflammatory cytokines on the relationship among gut microbiota, skin microbiota, and asthma. The Strengthening the Reporting of Observational Studies in Epidemiology Using MR checklist was completed for this observational study (**Supplementary Table S2**) (25). The GWAS data were obtained from publicly available datasets published between 2020 and 2023, and all data analyses were conducted in 2024.

### 2.2 Data sources

#### 2.2.1 Source of GWAS data on gut microbiota

A GWAS dataset that included 7738 Dutch Microbiome Project participants whose microbiota data were quality-controlled with LifeLines (26) was used. A total of 207 taxa (five phyla, 10 classes, 13 orders, 26 families, 48 genera, and 105 species) and 205 pathways representing microbial composition and function were included in the subsequent analysis.

#### 2.2.2 Source of GWAS data on skin microbiota

Data on skin microbiota in this study were obtained from the participants of two cross-sectional, population-based German cohorts, KORA FF4 (individuals = 324) and PopGen (individuals = 273), with a total of 1656 skin samples (27). Skin samples were taken from dry (dorsal and volar forearm [PopGen]), moist (antecubital fossa [KORA FF4 and PopGen]), and sebaceous (retro auricular fold [KORA FF4] and forehead [PopGen]) skin microenvironments. Microbial community profiles were obtained from the sequencing of the V1–V2 regions from the 16 S ribosomal RNA gene. Genome-wide association analyses were conducted on the univariate relative abundances of individual bacteria (amplicon sequence variants [ASVs]) and 79 nonredundant taxonomic groups ranging from genus to phylum levels.

#### 2.2.3 Source of GWAS data on metabolites

The summary statistics of plasma metabolomics were acquired from the GWAS Catalog (https://www.ebi.ac.uk/gwas/) with the study accession numbers GCST90199621–GCST90201020. The latest study included 1091 plasma metabolites and 309 metabolite ratios from 8299 European individuals and involved 8299 samples and approximately 150 000 single-nucleotide polymorphism (SNP) loci (28). The above study included 850 known metabolites out of 1091 plasma metabolites, which could be divided into eight broad metabolic groups: lipids (395), amino acids (210), xenobiotics (130), nucleotides (33), cofactors and vitamins (31), carbohydrates (22), peptides (21), and energy (8). The remaining metabolites were partially characterized molecules (21) and unknown (220).

#### 2.2.4 Source of GWAS data on WBC counts

Effect estimates for SNPs associated with WBC subtype counts, which included basophils, eosinophils, monocytes, lymphocytes, and neutrophils, were obtained from the Blood Cell Consortium meta-analysis involving 746 667 participants, including 184 424 individuals of non-EUR descent (29).

#### 2.2.5 Source of immunity-wide GWAS data

The GWAS summary statistics for each immune trait used for cellular subpopulation analyses are publicly available from the GWAS Catalog (accession numbers GCST0001391–GCST0002121) (30). A total of 731 immunophenotypes, including absolute cell (AC) counts (n = 118), median fluorescence intensities (MFI) reflecting surface antigen levels (n = 389), morphological parameters (MPs) (n = 32), and relative cell (RC) counts (n = 192), were included. Specifically, the MFI, AC, and RC features contained B cells, CDCs, mature stages of T cells, monocytes, myeloid cells, TBNK (T, B, and natural killer cells), and Treg panels, whereas the MP feature contained cDC and TBNK panels. The original GWAS on immune traits was performed by using data from 3757 individuals through flow cytometry with the Sardinian founder population (31).

#### 2.2.6 Source of GWAS data on circulating inflammatory proteins

The summary statistics of plasma metabolomics were acquired from the GWAS Catalog (https://www.ebi.ac.uk/gwas/) under the study accession numbers GCST90274758–GCST90274848. The study conducted a genome-wide protein quantitative trait locus study of 91 plasma proteins on 14824 participants measured by using the Olink Target platform (32).

#### 2.2.7 Source of GWAS data on inflammatory cytokines

GWAS data for 41 inflammatory cytokines were collected from the University of Bristol (https://data.bris.ac.uk/data/dataset). They included three Finnish cohort studies (N = 8,293): the Cardiovascular Risk in Young Finns Study, FINRISK1997, and FINRISK2002 (33, 34).

#### 2.2.8 Source of GWAS data on asthma

The GWAS summary-level data of asthma were extracted from FinnGen Biobank by the IEU open GWAS project, which included 42 163 European-descent cases and 202 399 European-descent controls.

### 2.3 Instrumental variable selection

In MR analysis, Instrumental variables (IVs) were utilized as mediators between exposure factors and outcomes to explore the causal relationship between exposure and outcomes. IVs are generally genetic variations, among which SNPs are the most used.

Strict quality control was performed on the SNPs to select valid IVs for MR analysis. Three basic assumptions of IVs must be satisfied. 1) Relevance assumption: IVs are related to the exposure studied. 2) Independence assumption: IVs are not associated with confounding factors. 3) Exclusion assumption: IVs do not directly affect the outcome. They can only affect the outcome by influencing exposure factors.

Three core assumptions were followed to identify the IVs needed:

1. The genome-wide significance threshold of *P* < 5 × 10^−8^ is used as a potential tool variable related to each exposure trait. When few whole-genome significance loci were found in the original GWAS results (35, 36), a loose *P* value of 1 × 10^−5^ was used to select SNPs associated with gut microbiota and plasma metabolomics, and a *P* value of 5 × 10^−5^ was applied to select SNPs associated with skin microbiota as candidate IVs.
2. SNPs related to outcome variables were excluded (*P* < 0.05).
3. Independent SNPs were then clumped to a linkage disequilibrium threshold of r^2^ < 0.001 in accordance with the 1000 Genomes reference panel.
4. The MR–Egger and MR pleiotropy residual sum and outlier (MR–PRESSO) test was used to assess horizontal pleiotropy. The pleiotropy effect was eliminated by removing outliers (37).
5. The strength of the selected SNPs was assessed by using the F-statistic, and variants with F statistic < 10 were excluded from the analysis to avoid weak instrumental bias. The F statistic formula is F = [R2 × (n − k − 1)]/[k × (1 − R2)], where R2 is the portion of the exposure variance explained by the IVs, n is the sample size, and k represents the number of IVs (38).
6. Steiger filtering was used to remove variants with evidence of a stronger association with the outcome than its association with the exposure.

### 2.4 Statistical analysis

#### 2.4.1 Two-sample MR analysis

A two-sample MR was conducted to assess the causal relationship among gut microbiota, skin microbiota, plasma metabolites, WBC subtype counts, immune cell traits, inflammatory proteins, inflammatory cytokines, and asthma. Various methods were employed to estimate MR effects, ensuring robustness. Inverse–variance weighted (IVW) served as the primary approach (39) and was supplemented by the MR–Egger (40), weighted mode (41), and weighted median (42) methods, each tailored to different assumptions of instrument validity.

Sensitivity analyses were performed to verify the robustness of causality and thus determine whether heterogeneity and pleiotropy within IVs could bias the MR results. Heterogeneity testing was performed by using the MR–Egger and IVW methods. Cochrane’s Q statistic was utilized to assess heterogeneity among genetic instruments, with *P *> 0.05 indicating the absence of significant heterogeneity. The MR–Egger regression equation was employed to evaluate the horizontal pleiotropy of genetic instruments, with *P *> 0.05 suggesting the absence of horizontal pleiotropy (43). Furthermore, a powerful method, MR–PRESSO (37), in the MR–PRESSO package was utilized to exclude possible horizontal pleiotropic outliers that could substantially affect estimation results. Steiger filtering (44) was conducted to remove variants with evidence of a stronger association with the outcome than its association with the exposure. To test the robustness of our MR results, we performed a leave-one-out sensitivity analysis, sequentially removing each SNP and recalculating the causal estimates.

Furthermore, in consideration of the potential chance to increase the overall type I error during multiple comparisons, false discovery rate (FDR) correction was implemented on the primary IVW results by using the Benjamini–Hochberg procedure. A significance threshold of FDR < 0.1 indicates a significant association, whereas *P_IVW_* < 0.05 but FDR > 0.1 implies a suggestive association (45). The source code used to analyze experiment results is publicly available at https://www.frontiersin.org/journals/immunology/articles/10.3389/fimmu.2025.1436888/abstract#supplementary-material.

All analyses were performed by using two-sample MR (version 0.6.0), MR (version 0.8.0), and MRPRESSO package (1.0) in R Software 4.3.3 (https://www.R-project.org).

#### 2.4.2 Reverse MR analysis

Reverse MR analysis was performed to investigate whether asthma had a causal effect on gut microbiota and skin microbiota (PIVW < 0.05). In this context, asthma SNPs were regarded as IVs, asthma as exposure, and gut, and skin microbiological features as outcomes. The procedure for reverse MR analysis was similar to that used for MR analysis.

#### 2.4.3 Metabolic pathway analysis

The HMDB IDs of known metabolites were retrieved from The Human Metabolome Database (https://hmdb.ca/) to identify known plasma metabolites (*P*_IVW_ < 0.05), and enrichment analysis was conducted on the metabolic pathways associated with these metabolites by using MetaboAnalyst 5.0 (https://www.metaboanalyst.ca/). The pathway libraries selected for this analysis were the Small Molecule Pathway Database and Kyoto Encyclopedia of Genes and Genomes. The enrichment method employed was the hypergeometric test, and the significance level for metabolic pathway analysis was set at 0.01.

#### 2.4.4 Linkage disequilibrium score regression analysis

Bivariate LDSC was performed by using GWAS summary statistics to show the genetic correlation among causal gut microbiota, causal skin microbiota, and asthma (46). χ^2^ statistics based on SNPs were regressed through LDSC regression to determine the heritability of a single trait and coheritability of two traits, which can identify whether confounding factors were present in MR analysis.

#### 2.4.5 Mediation analysis

Mediation analysis can help explore potential mechanisms through which exposure affects outcomes. MR can be used to improve causal inference for mediation analysis (24). The mediation analysis in this study focused on asthma-related gut microbiota, skin microbiota, plasma metabolites, immune cell traits, inflammatory proteins, and inflammatory cytokines. The causal effect of exposure on the outcome (beta of IVW is c) was calculated before the two-step MR analysis was performed.

The mediation effect was calculated by using two-step MR as follows: mediation effect = beta (A) × beta (B). The total effect of gut microbiota on asthma was obtained through the previous two-sample MR and direct effect = (total effect − mediation effect). The mediation proportion was calculated by using the following formula: mediation proportion = (mediation effect/total effect) × 100%. The 95% confidence intervals (CI) for the mediation effects and proportions mediated were estimated by using the delta method.

## 3 Results

### 3.1 Causal effects of gut microbiota on asthma

**Figure 2:**
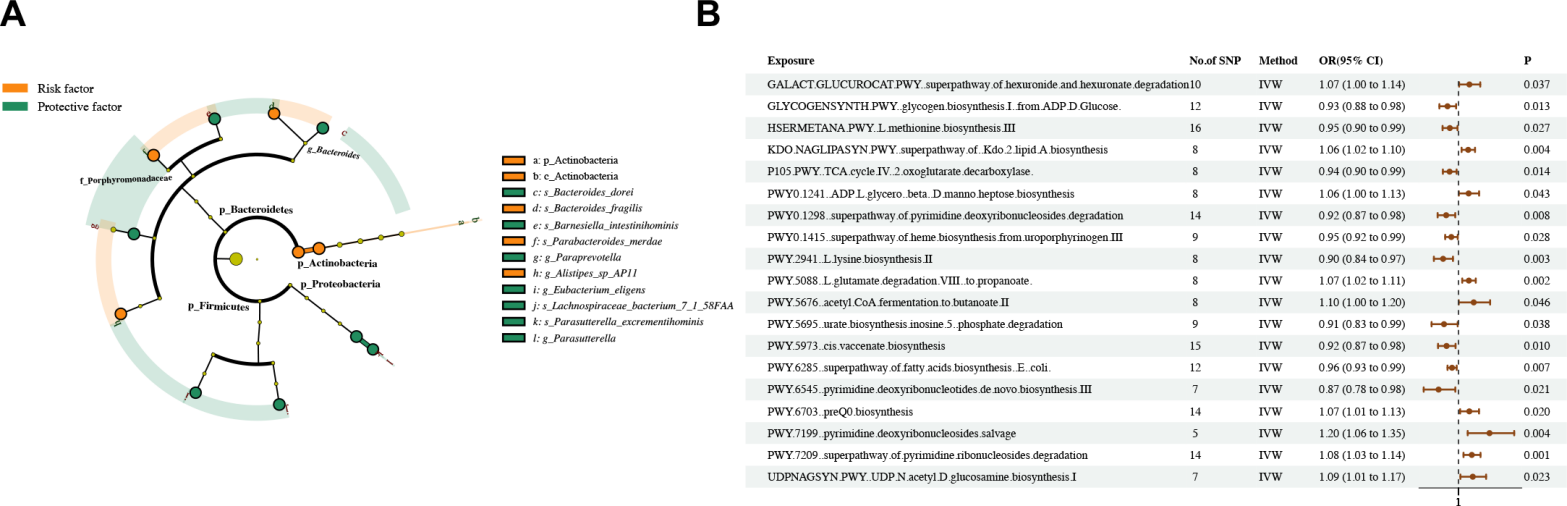
Causal estimates of MR between gut microbiota and asthma. **(A)** Estimates from the IVW analysis of gut microbiota abundance on asthma. **(B)** Estimates from the IVW analysis of gut bacterial pathway abundance on asthma.

Through two-sample MR analysis, we identified 31 suggestive associations between gut microbiota and asthma (*P*_IVW_ < 0.05, FDR > 0.1; including the abundances of 19 gut bacterial pathways and 12 gut microbiota) (**Figure 2**; **Supplementary Tables S3**–**5**).

The phylum Actinobacteria and class Actinobacteria in the phylum Actinobacteria had a positive causal relationship with asthma. The species *Parabacteroides merdae*, *Alistipes* sp *AP11*, and *Bacteroides fragilis* in the phylum Bacteroidetes had a positive causal relationship with asthma. By contrast, the genus *Paraprevotella*, species *Barnesiella intestinihominis*, and species *Bacteroides dorei* in the phylum Bacteroidetes had a negative causal relationship with asthma. The genus *Parasutterella* and species *Parasutterella excrementihominis* in the phylum Proteobacteria had a negative causal relationship with asthma. The species *Eubacterium eligens* and *Lachnospiraceae bacterium 7 1 58FAA* in the phylum Firmicutes had a negative causal relationship with asthma.

Dangerous and protective bacteria are partly independent and intertwined. The species *Bacteroides dorei* and *Bacteroides_fragilis* belong to the genus *Bacteroides*. The species *Barnesiella intestinihominis* and *Parabacteroides merdae* belong to the family Porphyromonadaceae. The above strains are members of the phylum Bacteroidetes together with the genus *Paraprevotella* and species *Alistipes* sp *AP11*. Meanwhile, the phyla Proteobacteria, Firmicutes, and Actinobacteria have an internal unified direction of influence on asthma (**Figure 2A**). The abundances of nine gut bacterial pathways had a positive causal relationship with asthma, whereas those of 10 gut bacterial pathways had a negative causal relationship (**Figure 2B**).

Sensitivity analysis further verified the robustness of the MR results (**Supplementary Table S6**). The Q statistics showed that only the species *Bacteroides dorei* had heterogeneity, whereas the remaining species did not show evidence of heterogeneity. Furthermore, the results of MR–Egger regression and MR–PRESSO did not reveal horizontal pleiotropy (dual validation was conducted on the abundances of 18 gut bacterial pathways and 11 gut microbiota). We did not find any reverse causality on the basis of the MR–Steiger test.

We conducted reverse MR and found a negative causal relationship between asthma and the family Streptococcaceae (odds ratio [OR] = 0.854, 95% CI [0.742–0.982], *P* = 0.027), genus *Streptococcus* (= 0.831, 95% CI [0.721–0.957], *P* = 0.010), family Oscillospiraceae (OR = 0.857, 95% CI [0.769–0.955], *P* = 0.005), genus *Oscillibacter* (OR = 0.857, 95% CI [0.769–0.955], *P* = 0.005), and species *Oscillibacter unclassified* (OR = 0.850, 95% CI [0.764–0.947], *P* = 0.003) (**Supplementary Tables S7**–**10**). Interestingly, all the above strains belonged to the phylum Firmicutes, which was negatively associated with asthma in forward and reverse analyses.

We then performed bivariate LDSC and identified a strong positive genetic correlation between the species *Alistipes* sp *AP11* and asthma (rg = 0.06, *P*= 0.619) (**Supplementary Tables S11**).

### 3.2 Causal effects of skin microbiota on asthma

**Figure 3:**
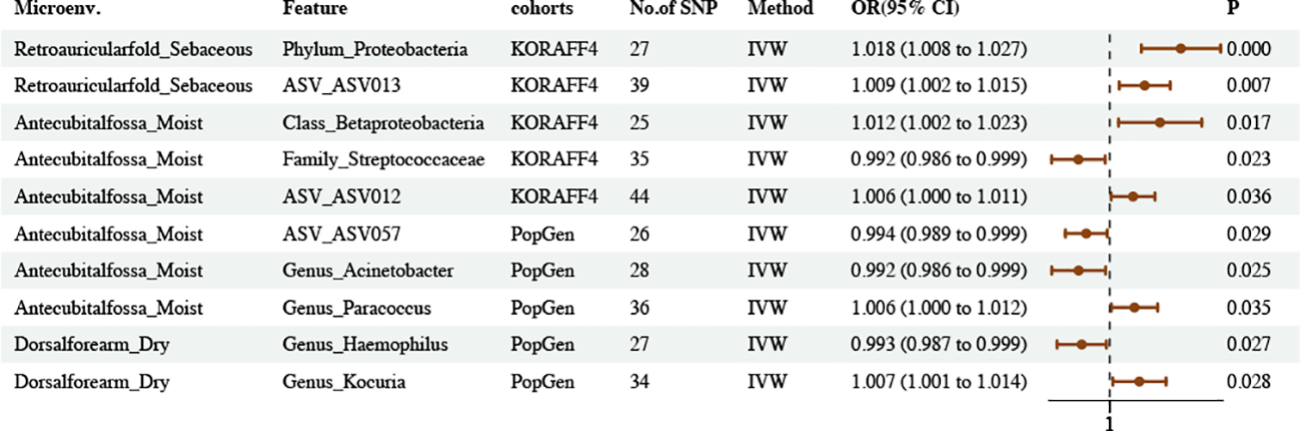
Causal estimates of MR between skin microbiota and asthma. Estimates from the IVW analysis of skin microbiota on asthma.

The results obtained through the IVW method suggested 10 causal relationships between skin microbiota and asthma (*P*_IVW_ < 0.05, FDR > 0.1) (**Figure 3**; **Supplementary Tables S12**–**14**).

We found that the phylum Proteobacteria and ASV ASV013 in skin samples taken from a sebaceous (retro auricular fold [KORA FF4]) skin microenvironment had a positive causal relationship with asthma. The class Betaproteobacteria and ASV ASV012 in skin samples taken from a moist (antecubital fossa [KORA FF4]) skin microenvironment had a positive causal relationship with asthma. The genus *Paracoccus* in skin samples taken from a moist (antecubital fossa [PopGen]) skin microenvironment had a positive causal relationship with asthma. The genus *Kocuria* in skin samples taken from a dry (volar forearm [PopGen]) skin microenvironment had a positive causal relationship with asthma.

The family Streptococcaceae in skin samples taken from a moist (Antecubital fossa KORAFF4]) skin microenvironment had a negative causal relationship with asthma. The ASV ASV057 and genus *Acinetobacter* in skin samples taken from a moist (Antecubital fossa [PopGen]) skin microenvironment had a negative causal relationship with asthma. The genus *Haemophilus* in skin samples taken from a dry (dorsal forearm [PopGen]) skin microenvironment had a negative causal relationship with asthma.

We performed reverse MR and found no causal relationship between asthma and skin microbiota. Sensitivity analysis further verified the robustness of the MR results. Furthermore, MR–Egger regression and MR–PRESSO did not reveal horizontal pleiotropy. We did not find any reverse causality on the basis of the MR–Steiger test (**Supplementary Table S15**).

### 3.3 Causal effects of plasma metabolites on asthma

**Figure 4:**
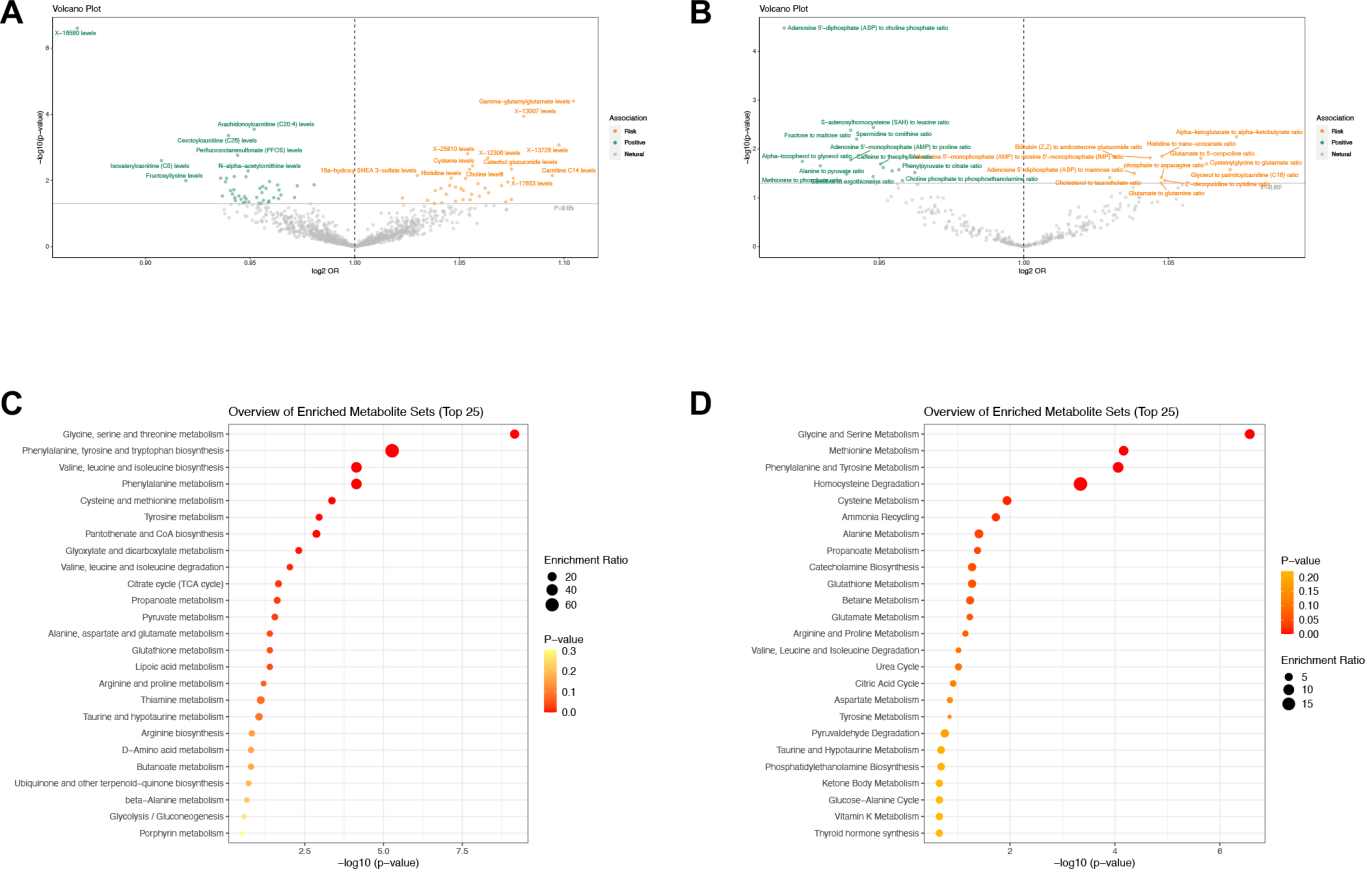
Causal estimates of MR between plasma metabolites and asthma. **(A)** Volcano plots of the IVW MR for the associations between plasma metabolites levels and asthma. **(B)** Volcano plots of the IVW MR for the associations between metabolic ratios and asthma. **(C)** The enrichment pathways of metabolites in KEGG. **(D)** The enrichment pathways of metabolites in SMPDE database.

The results obtained on the basis of the IVW method suggested 108 causal relationships between plasma metabolomics and asthma (*P*_IVW_ < 0.05, corresponding to 81 unique plasma metabolite levels and 27 unique metabolic ratios) (**Figures 4A, B**; **Supplementary Figure S1**, **Supplementary Tables S16**–**18**). Plasma metabolites included amino acids (17), carbohydrates (2), cofactors and vitamins (3), lipids (30), nucleotides (1), partially characterized molecules (1), peptides (3), xenobiotics (4), and unknown (20).

However, following FDR correction, only four results maintained significant negative or positive causal relationships with asthma. Adenosine-5′-diphosphate-to-choline phosphate ratio maintained a significant negative causal relationship with asthma (OR = 0.9171, 95% CI [0.8804–0.9554], *P* = 0.00003307, FDR = 0.0186). X-16580 levels maintained a significant negative causal relationship with asthma (OR = 0.8674, 95% CI [0.8217–0.9155], *P* = 0.00000025, FDR = 0.0003). X-13007 levels maintained a significant positive causal relationship with asthma (OR = 1.0808, 95% CI [1.0389–1.1242], *P* = 0.00011440, FDR = 0.0400). Gamma-glutamyl glutamate levels maintained a significant positive causal relationship with asthma (OR = 1.1045, 95% CI [1.0534–1.1581], *P* = 0.00003978, FDR = 0.0186).

Sensitivity analysis further verified the robustness of the MR results. The Q statistics showed that nine plasma metabolites had heterogeneity, whereas the rest did not show evidence of heterogeneity. Furthermore, the results of MR–Egger regression and MR–PRESSO did not reveal horizontal pleiotropy (dual validation using 72 plasma metabolite levels and 23 metabolic ratios). We did not find any reverse causality on the basis of the MR–Steiger test (**Supplementary Table S19**).

We queried the HMDB IDs for the 81 known metabolites associated with asthma and conducted metabolic pathway enrichment analysis on 51 identifiable compounds. The enrichment results highlighted the metabolic pathways of glycine, serine and threonine metabolism; phenylalanine, tyrosine, and tryptophan biosynthesis; and methionine metabolism (**Figures 4C, D**; **Supplementary Table S20**).

### 3.4 Causal effects of WBC subtype counts and immune cell traits on asthma

**Figure 5:**
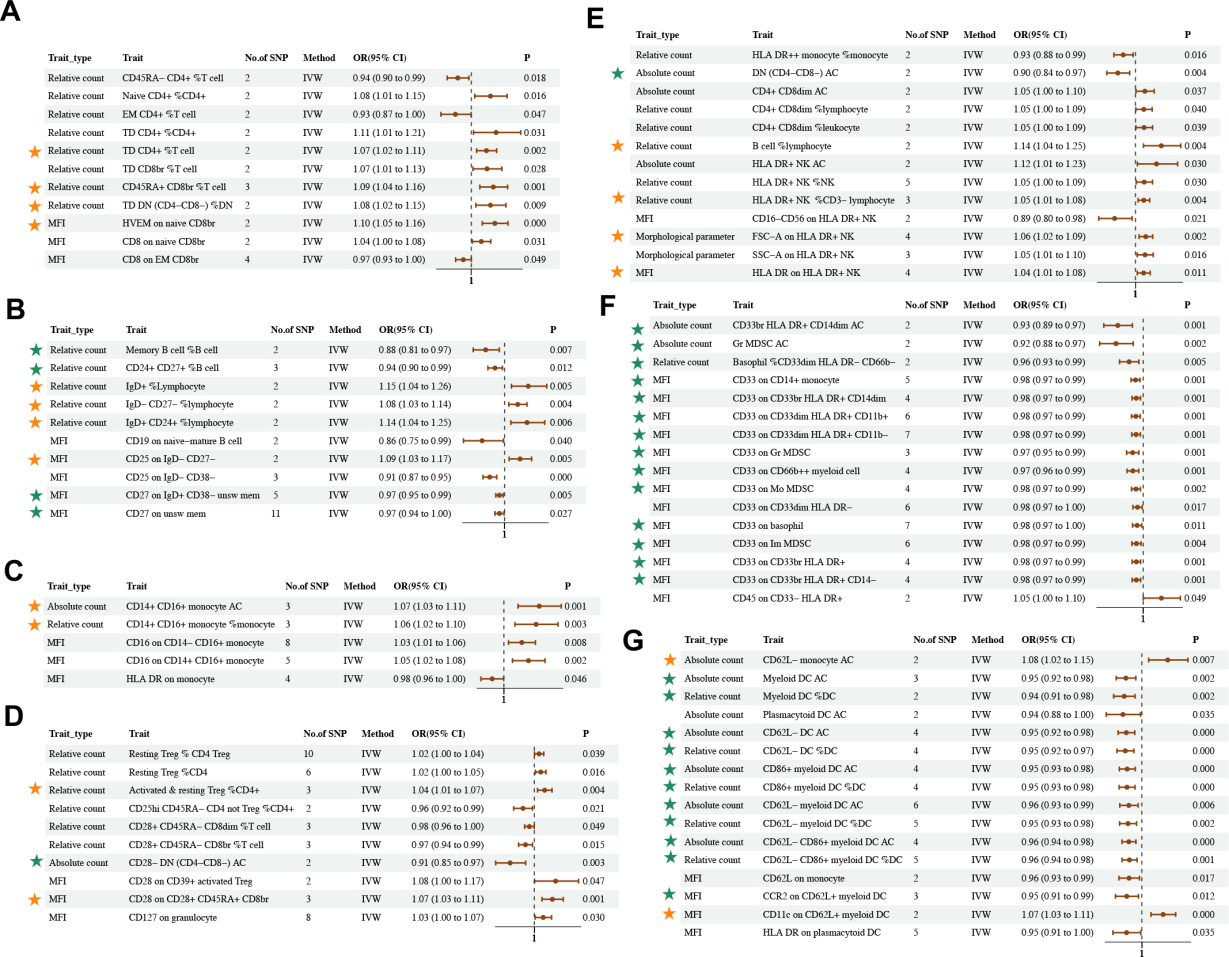
Causal estimates of MR between immune cell traits and asthma. Estimates from the IVW analysis of immune cell traits on asthma. Immune cell traits included **(A)** Maturation stages of T cell **(B)** B cell **(C)** Monocyte **(D)** Treg **(E)** TBNK **(F)** Myeloid cell **(G)** cDC. The results marked with green stars maintained significant negative causal relationship with asthma. The results marked with orange stars maintained significant positive causal relationship with asthma.

The IVW method revealed 35 associations between 81 unique immune cell traits and asthma (*P*_IVW_ < 0.05) (**Figure 5**; **Supplementary Tables S21**–**24**). Immune cell traits included B cells (10), cDC (16), maturation stages of T cells (11), monocytes (5), myeloid cells (16), TBNKs (13), and Tregs (10) (**Supplementary Table S25**). In the results of a further step following FDR correction, those marked with stars indicated the maintenance of significant negative or positive causal relationships with asthma. The MR results remained stable in sensitivity analyses, suggesting the absence of significant heterogeneity and horizontal pleiotropy (**Supplementary Table S26**).

Although bivariate LDSC analysis identified a strong negative genetic association between eosinophil cell count and asthma, the MR analysis of eosinophil cell count in asthma identified heterogeneity and pleiotropy, which affected the results of causality (**Supplementary Table S11**).

### 3.5 Causal effects of inflammatory proteins and cytokines on asthma

**Figure 6:**
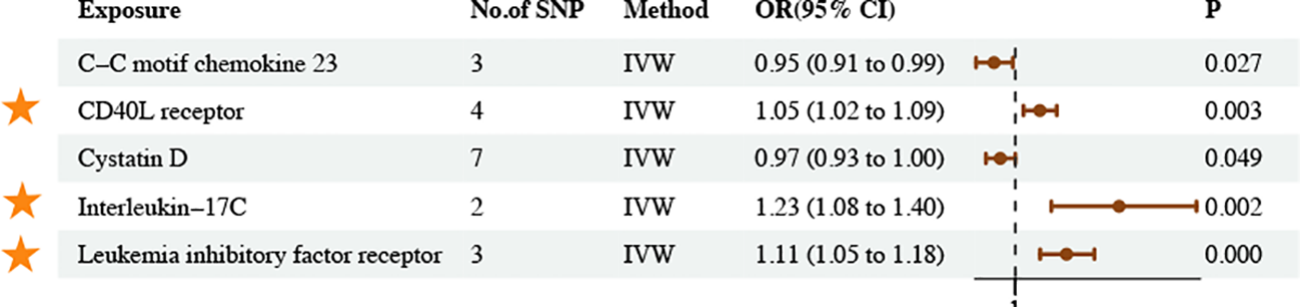
Causal estimates of MR between inflammatory proteins and asthma. Estimates from the IVW analysis of inflammatory proteins on asthma. The results marked with orange stars maintained significant positive causal relationship with asthma.

**Figure 7:**
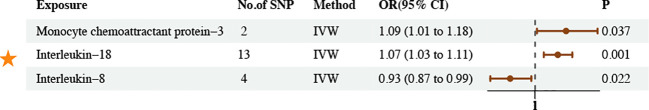
Causal estimates of MR between inflammatory cytokines and asthma. Estimates from the IVW analysis of inflammatory cytokines on asthma. The results marked with orange stars maintained significant positive causal relationship with asthma.

The IVW method revealed five associations between inflammatory proteins and asthma and three associations between inflammatory cytokines and asthma (*P*_IVW_ < 0.05) (**Figures 6**, **7**; **Supplementary Tables S27**–**30**). Following FDR correction, four results maintained a significant positive causal relationship with asthma. CD40L receptor levels maintained a significant positive causal relationship with asthma (OR = 1.05, 95% CI [1.02–1.09], *P* = 0.003, FDR = 0.0588). IL-17C levels maintained a significant positive causal relationship with asthma (OR = 1.23, 95% CI [1.08–1.40], *P* = 0.002, FDR = 0.0554). Leukemia inhibitory factor receptor levels maintained a significant positive causal relationship with asthma (OR = 1.11, 95% CI [1.05–1.18], *P* = 0.000, FDR = 0.0057). IL-18 levels maintained a significant positive causal relationship with asthma (OR = 1.07, 95% CI [1.03–1.11], *P* = 0.001, FDR = 0.0518). The MR results remained stable in the sensitivity analyses, suggesting the absence of significant heterogeneity and horizontal pleiotropy (**Supplementary Table S31**).

### 3.6 Mediation analysis results

We based our findings on asthma-associated gut microbiota, skin microbiota, metabolites, cells, proteins, and cytokines in two previously identified samples of MR to explore the potential mechanisms for the occurrence and development of asthma. We performed two-step MR analysis with plasma metabolites, immune cell traits, and inflammatory proteins and cytokines as mediator variables.

**Figure 8:**
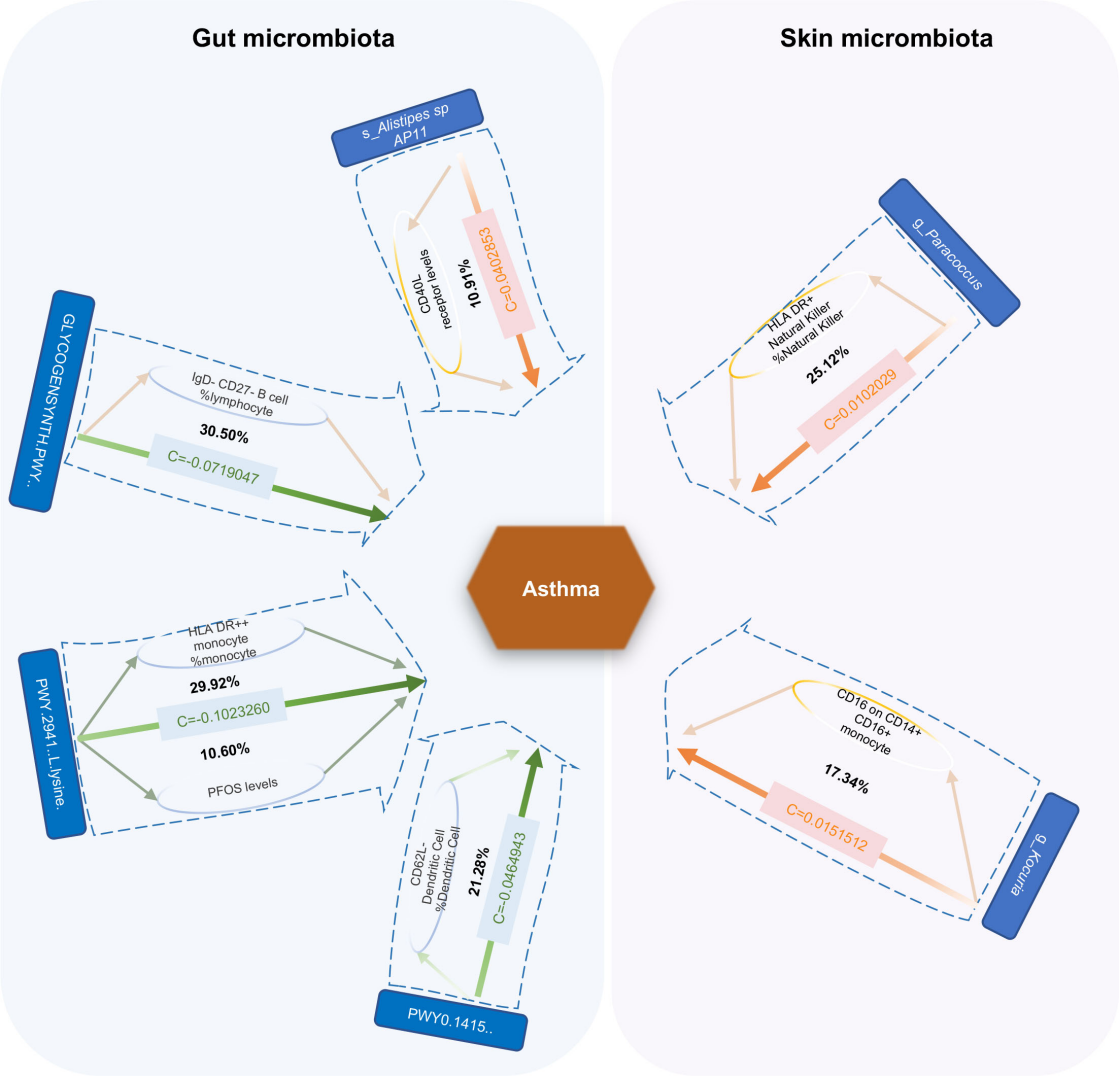
Mediation effect of gut microbiota or skin microbiota on asthma via plasma metabolites, immune cell traits, and inflammatory proteins. The green lines represent protective effects, and the orange lines represent risk effects. The mediation proportion is marked in the middle of the box. PFOS Perfluoro octane sulfonate; c Total effect; GLYCOGENSYNTH.PWY.glycogen.biosynthesis.I.from.ADP.D.Glucose; PWY.2941.L.lysine.biosynthesis.II; PWY0.1415.superpathway.of.heme.biosynthesis.from.uroporphyrinogen.III.

We found seven mediators that were significant intermediate variables linking gut microbiota or skin microbiota with asthma. The results showed that three gut bacterial pathways play a protective role in asthma through the mediation of IgD–CD27–B cell %lymphocyte, CD62L-DC %DC, HLA DR++ monocyte %monocyte, and perfluoro octane sulfonate levels. On the other hand, three bacteria played a predisposing role in asthma through the mediating effects of HLA DR+ natural killer %natural killer, and CD16 on CD14+ CD16+ monocyte and CD40L receptor levels (**Figure 8**; **Supplementary Table S32**).

## 4 Discussion

In this study, we first assessed the causal relationship among gut microbiota, skin microbiota, plasma metabolome, WBC subtype counts, immune cell traits, circulating inflammatory proteins, inflammatory cytokines, and asthma by using MR analysis. We found potential causal associations among 31 gut bacterial features (abundances of 19 bacterial pathways and 12 microbiota), 10 skin bacterial features, 108 plasma metabolites (81 metabolites and 27 ratios), 81 immune cell traits, five circulating inflammatory proteins, three inflammatory cytokines, and asthma. Furthermore, the results of our mediation analysis supported the mediating effects of one plasma metabolite, five immunophenotypes, and one inflammatory protein on gut or skin microbiota in asthma pathogenesis.

A previous study found that the phylum Firmicutes showed significantly low abundance in children with asthma, and its dysbiosis may be associated with an increased risk of asthma (10). The negative association between the species *Eubacterium eligens* and *Lachnospiraceae bacterium 7 1 58FAA* in the phylum Firmicutes and asthma in our study was consistent with this previously reported finding. Another metabolomics-based study performed a comparative analysis on stool samples from children with asthma and healthy children aged 4–7 years. It found that children with allergic airway illnesses tended to have a considerably lower abundance of Firmicutes than those without. These results suggested that childhood rhinitis and asthma may be caused by a reduction in certain gut microbes in the phylum Firmicutes that are involved in the up-regulation of fecal amino acids (47). Therefore, through our study, we can infer whether the specific gut microbes in the phylum Firmicutes showing reductions may be the species *Eubacterium eligens* and *Lachnospiraceae bacterium 7 1 58FAA*. Data from the Canadian Healthy Infant Longitudinal Development Study demonstrated that the relative abundance of *Rothia* (phylum Actinobacteria) was considerably reduced in the gut microbiome of infants at risk for asthma in the first 100 days of life (48). We found that phylum Actinobacteria and class Actinobacteria in phylum Actinobacteria had a positive causal relationship with asthma likely due to the role of other bacteria in Actinobacteria. A positive association was found between *Bacteroides fragilis* colonization and asthma predictive index (49), and we obtained findings consistent with this result. The relative abundances of Bacteroidetes increased in the symptomatic eosinophilic asthma group (49). In our study, the effects of bacteria in Bacteroidetes on asthma are complicated, and the overall effects on asthma are unknown.

In healthy, but not in atopic, subjects, the relative abundance of Acinetobacter species was associated with the expression of anti-inflammatory molecules by PBMCs. Moreover, healthy subjects exhibited a robust balance between anti-inflammatory and Th1/Th2 gene expression, which was related to the composition of the skin microbiota. In cell assays and a mouse model, Acinetobacter species induced strong Th1 and anti-inflammatory responses by immune and skin cells and protected against allergic sensitization and lung inflammation through the skin. Acinetobacter species in the skin microbiota were found to protect against allergic sensitization and inflammation (19). These findings are similar to ours.

Patients with asthma were characterized by high levels of methionine, glutamine, and histidine and low levels of formate, methanol, acetate, choline, *O*-phosphocholine, arginine, and glucose (50). Similarly, we found that histidine (OR = 1.046, 95% CI [1.012–1.082], *P* = 0.009) and glutamine (OR = 1.053, 95% CI [1.013–1.094], *P* = 0.009) levels had a positive causal relationship with asthma. Metabolic pathway enrichment analysis on 51 identified compounds included methionine (Raw *P*val = 0.0000682, FDR = 0.00284) and histidine (Raw *P*val = 0.56, FDR = 1) metabolism.

Asthma is a highly heterogeneous disease with numerous endotypes based on discrete pathophysiological mechanisms. The complexity of asthma is due to the involvement of multiple cell types, including tissue-resident ILCs and other innate immune cells, such as bronchial epithelial cells, DCs, macrophages, and eosinophils (51). Numerous myeloid cells play a crucial role in asthma pathogenesis (52). Meanwhile, allergic asthma is the most common asthma phenotype (53). A considerable proportion of myeloid DCs rapidly disappear from circulation following allergen inhalation, suggesting that the margination of circulating myeloid DCs, as well as their recruitment into the airway mucosa, is an important feature of the immune response to inhaled allergens (54). Our findings are consistent with the above results. Eosinophils, a type of immune cell, play a critical role in the development and progression of asthma (55). Eosinophils are more than a marker of type 2 high asthma (14). Specifically, our bivariate LDSC analysis identified a strong negative genetic association between eosinophil cell counts and asthma.

The CD40 receptor and its ligand CD40L is one of the most critical molecular pairs of stimulatory immune checkpoints. Nonhematopoietic cells expressing CD40 can also engage CD40L and trigger a proinflammatory response (56). IL-17C has been known to participate in allergic inflammation. It is produced by distinct cellular sources and an essential autocrine cytokine that regulates innate epithelial immune responses (57). IL-17C is a member of the IL-17 family that is selectively induced in epithelia by bacterial challenge and inflammatory stimuli. IL-17C stimulates epithelial inflammatory responses, including the expression of proinflammatory cytokines, chemokines, and antimicrobial peptides, which are similar to those induced by IL-17A and IL-17F (58, 59). In addition, IL-18 may act as a potential mediator in the causal relationship between adult-onset asthma and ulcerative colitis (60). IL-18 expression in the lamina propria in biopsies from subjects with asthma did not differ from that in biopsies from controls but decreased in the epithelium (61). Recent work identified the association of IL-18 with the pathogenesis of asthma, wherein increased IL-18 expression was found in the serum of patients. Furthermore, IL-18 polymorphisms have been reported to be associated with susceptibility to asthma, suggesting that IL-18 may be therapeutically relevant to asthma (62). The above findings are similar to the results of our study.

Finally, we identified a few gut or skin bacteria on the basis of plasma metabolites, immune phenotype, and inflammatory proteins involved in the pathogenesis of asthma. Related research remains scarce, and additional studies are needed on the role of the above factors in asthma in the future. This study has certain limitations. First, under the currently set screening conditions, we obtained few SNPs for some immune cells. This situation may have led to a bias in the results. Second, given that our population data originated from individuals of European ancestry, the generalizability of our research results to other populations is limited. Third, asthma type analysis will provide a clear information basis for subsequent clinical and experimental studies. The validity of our study’s results should be further confirmed through additional experimental and clinical studies.

## 5 Conclusion

Our MR study identified 31 gut bacterial features (abundances of 19 bacterial pathways and 12 microbiota), 10 skin bacterial features, 108 plasma metabolites (81 metabolites and 27 ratios), 81 immune cells, five circulating inflammatory proteins, and three inflammatory cytokines involved in asthma. Moreover, the results of our mediation analysis supported the mediating effects of perfluoro octane sulfonate (PFOS, a plasma metabolite), IgD–CD27–B cells, CD62L-DCs, HLA DR++ monocytes, HLA DR+ natural killer cells, and CD16 on CD14+ CD16+ monocytes (five immunophenotypes), and CD40L receptor (an inflammatory protein) on gut or skin microbiota in asthma pathogenesis. Our findings contribute to the study of asthma mechanisms.

## Data Availability

The datasets presented in this study can be found in online repositories. The names of the repository/repositories and accession number(s) can be found in the article/**Supplementary Material**.
