# Corrigendum: Effect of the gut microbiome, skin microbiome, plasma metabolome, white blood cells subtype, immune cells, inflammatory proteins, and inflammatory cytokines on asthma: a two-sample Mendelian randomized study and mediation analysis

**DOI:** 10.3389/fimmu.2025.1598472

**Published:** 2025-05-07

**Authors:** Wenqian Guo, Er Hong, Han Ma, Ji Wang, Qi Wang

**Affiliations:** ^1^ School of Traditional Chinese Medicine, Southern Medical University, Guangzhou, China; ^2^ National Institute of Traditional Chinese Medicine Constitution and Preventive Medicine, Beijing University of Chinese Medicine, Beijing, China; ^3^ Department of Respiratory Medicine, Ningbo Hospital of Traditional Chinese Medicine, Zhejiang University of Chinese Medicine, Ningbo, China; ^4^ The Second Affiliated Hospital of Henan University of Chinese Medicine, Zhengzhou, China

**Keywords:** asthma, gut microbiota, skin microbiota, plasma metabolites, immune cells, inflammatory proteins, inflammatory cytokines, Mendelian randomization

In the published article, there was an error in Affiliation 1. Instead of “College of Traditional Chinese Medicine, Southern Medical University, Guangzhou, China”, it should be “School of Traditional Chinese Medicine, Southern Medical University, Guangzhou, China”.

The authors apologize for this error and state that this does not change the scientific conclusions of the article in any way. The original article has been updated.

