# Supplementary figures and images for: Effect of the gut microbiome, skin microbiome, plasma metabolome, white blood cells subtype, immune cells, inflammatory proteins, and inflammatory cytokines on asthma: a two-sample Mendelian randomized study and mediation analysis
Source: Front Immunol. 2025 Mar 21;16:1436888. doi: 10.3389/fimmu.2025.1436888 (PMC11968350; doi:10.3389/fimmu.2025.1436888)

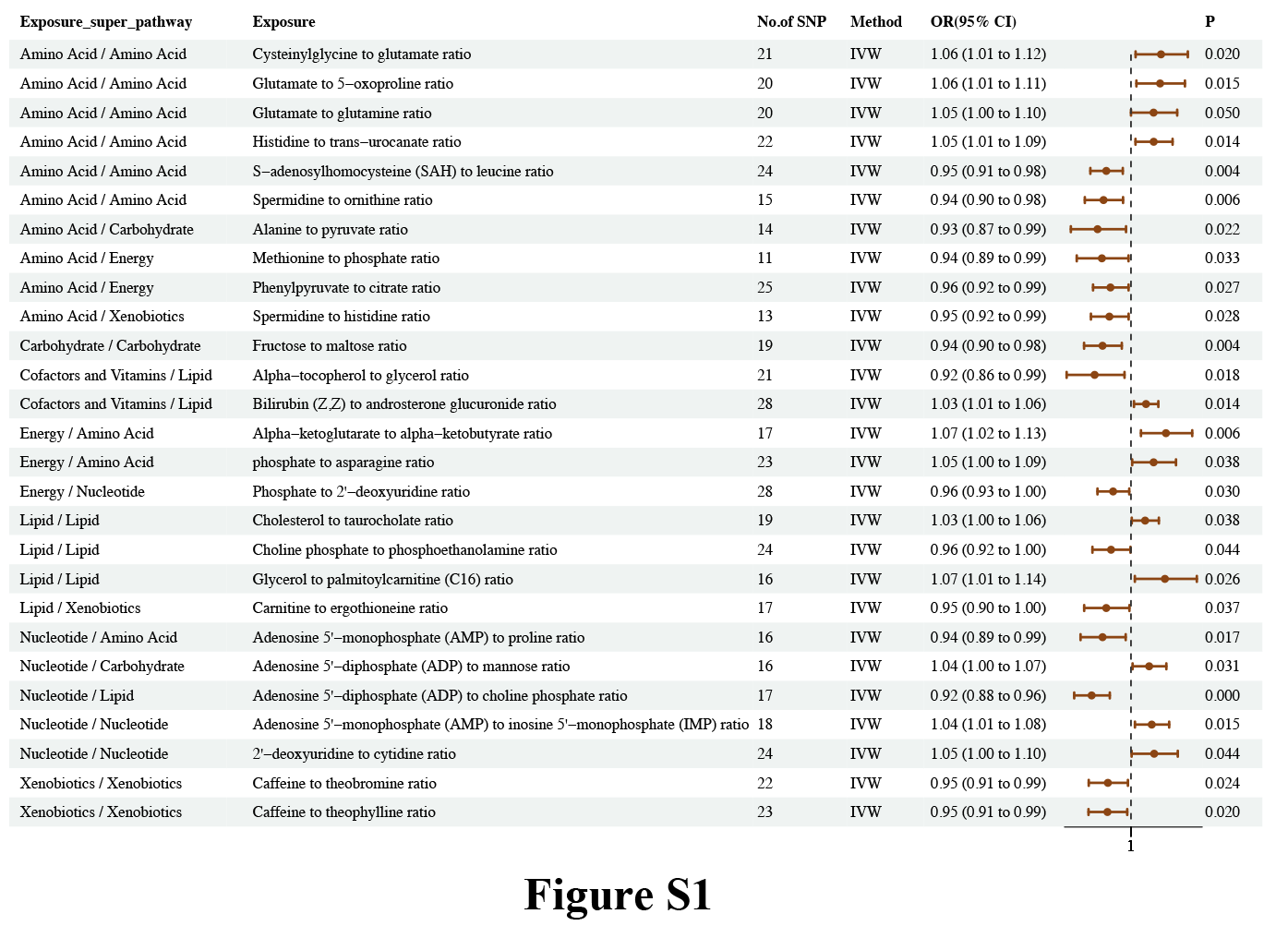

Supplement: Supplementary file 1 [file Image1.tif]
