# Supplementary material for: Effect of the gut microbiome, skin microbiome, plasma metabolome, white blood cells subtype, immune cells, inflammatory proteins, and inflammatory cytokines on asthma: a two-sample Mendelian randomized study and mediation analysis
Source: Front Immunol. 2025 Mar 21;16:1436888. doi: 10.3389/fimmu.2025.1436888 (PMC11968350; doi:10.3389/fimmu.2025.1436888)
Supplement: Supplementary file 3 [file DataSheet2.docx]

**R Scripts for Mendelian Randomization and Mediation Analysis**

library(TwoSampleMR)

library(MRPRESSO)

library(data.table)

#run mr analysis

mr_fun <- function(id_exposures, id_outcomes, p1=5e-8,

method_list = c("mr_ivw","mr_egger_regression","mr_weighted_median","mr_weighted_mode"),

rm_snps=NULL){

#1.read local exposure data

exposure_data <- read_exposure_data(id_exposures)

exposure_data <- clump_data(exposure_data, clump_p1 = p1, clump_r2=0.001, clump_kb=10000)

#remove some snps

if(is.null(rm_snps)){

exposure_data <- subset(exposure_data, !SNP %in% rm_snps)

}

#2.read online ieu outcome data

outcome_data <- read_outcome_data(id_outcomes, exposure_data$SNP)

#3.harmonise data

harmonise_data <- harmonise_data(exposure_data, outcome_data)

#4.run mr analysis

mr_res <- mr(harmonise_data, method_list = method_list)

#5.sensitivity analysis

het_res <- mr_heterogeneity(harmonise_data)

ple_res <- mr_pleiotropy_test(harmonise_data)

#6. run presso

presso_res <- mr_presso(harmonise_data$beta.outcome, harmonise_data$beta.exposure, harmonise_data$se.outcome, harmonise_data$se.exposure)

return(mr_res)

}

#read local data and run mr

path_to_cfb <- "path_to_pqtl"

path_to_migraine <- "ukb-saige-189.2.csv"

for(f in list.files(path_to_cfb, full.names = T)){

mr_fun(f, path_to_migraine) #forward

mr_fun(path_to_migraine, f) #reverse

}

mediation_fun <- function(id_exposure, id_mediation, id_outcome){

#1.run exposure to mediator mr

dat_exposure_mediator <- mr_fun(id_exposure, id_mediation, method_list = "mr_ivw")

#2.run exposure to outcome mr

dat_exposure_outcome <- mr_fun(id_exposure, id_outcome, method_list = "mr_ivw")

#3.run mediator to outcome mr

dat_mediator_outcome <- mv_fun(id_mediation, id_outcome, method_list = "mr_ivw")

#4.calculate mediation effect

a <- as.numeric(dat_exposure_mediator$b)

b <- as.numeric(dat_mediator_outcome$b)

c1 <- as.numeric(dat_exposure_outcome$b)

sa <<- as.numeric(dat_exposure_mediator$se)

sb <<- as.numeric(dat_mediator_outcome$se)

#5.calculat effect

total_effect <- c1

mediator_effect <- a * b

direct_effect <- c1 - IE

mediator_proportion <- IE / TE

}
